# Data for factor analysis of hydro-geochemical characteristics of groundwater resources in Iranshahr

**DOI:** 10.1016/j.dib.2018.05.039

**Published:** 2018-05-18

**Authors:** Hamed Biglari, Mehdi Saeidi, Kamaleddin Karimyan, Mohammad Reza Narooie, Hooshmand Sharafi

**Affiliations:** a Department of Environmental Health Engineering, School of Public Health, Gonabad University of Medical Sciences, Gonabad, Iran; b Department of Environmental Health Engineering, School of Health, Torbat Heydariyeh University of Medical Sciences, Torbat Heydariyeh, Iran; c Environmental Health Research Center, Kurdistan University of Medical Sciences, Sanandaj, Iran; d Department of Environmental Health Engineering, School of Public Health, Tehran University of Medical Sciences, Tehran, Iran; e Department of Environmental Health Engineering, School of Public Health, Iranshahr University of Medical Sciences, Iranshahr, Iran; f Students Research Committee, Kermanshah University of Medical Sciences, Kermanshah, Iran

**Keywords:** Hydro-geochemistry, Factor analysis, Correlation matrix, Groundwater resources, Iranshahr

## Abstract

Detection of Hydrogeological and Hydro-geochemical changes affecting the quality of aquifer water is very important. The aim of this study was to determine the factor analysis of the hydro-geochemical characteristics of Iranshahr underground water resources during the warm and cool seasons. In this study, 248 samples (two-time repetitions) of ground water resources were provided at first by cluster-random sampling method during 2017 in the villages of Iranshahr city. After transferring the samples to the laboratory, concentrations of 13 important chemical parameters in those samples were determined according to o water and wastewater standard methods. The results of this study indicated that 45.45% and 55.55% of the correlation between parameters has had a significant decrease and increase, respectively with the transition from warm seasons to cold seasons. According to the factor analysis method, three factors of land hydro-geochemical processes, supplying resources by surface water and sewage as well as human activities have been identified as influential on the chemical composition of these resources.The highest growth rate of 0.37 was observed between phosphate and nitrate ions while the lowest trend of − 0.33 was seen between fluoride ion and calcium as well as chloride ions. Also, a significant increase in the correlation between magnesium ion and nitrate ion from warm seasons to cold seasons indicates the high seasonal impact of the relation between these two parameters.

**Specifications Table**

**Table:** 

Subject area	Environmental sciences
More specific subject area	Chemistry
Type of data	Tables and figures
How data was acquired	The data were collected by 248 samples from groundwater sources during 2017 in the villages of Iranshahr county. The concentrations of 13 important chemical parameters in those samples, including temporary and permanent hardness, calcium and magnesium, iron, nitrate, alkalinity, sulfate and chloride were determined.
Data format	Raw, analyzed
Experimental factors	All water samples in polyethylene bottles were stored in a dark place at room temperature until them analysed.
Experimental features	In this study, all of mentioned parameters in abstract part were analyzed according to the standard methods for water and wastewater treatment handbook.
Data source location	Iranshahr county, Sistan and Baluchestan province, Iran
Data accessibility	Data are included in this article

**Value of the data**

• Considering that no study about the effects of climatic and anthropogenic effects on the quality changes of groundwater resources in a plain has been carried out so far in Iran, the present study was carried out on the key aquifer of Iranshahr plain. Thus, in case significant results are observed, this study will be carried out on other aquifers.
• The data of this study reflect that the quality of groundwater resources in the Iranshahr area is situated under the influence of seasonal conditions. In the warm seasons, the overall chemical quality of the groundwater has been better due to the lack of rainfall in these waters.
• The data of this study showed that it is better to use surface currents in seasons where surface currents exist to supply drinking or agriculture water so that complications and purification costs can be reduced.
• Based on the data of the present study, it can be represented that only a few chemical parameters can be examined to determine the process of qualitative changes of other chemical parameters of water and there is no need to measure and monitor all chemical parameters of groundwater resources in each season.
• According to the dat of this study, there is also a meaningful relationship between qualitative changes of chemical parameters of water in each hot and cold seasons.
• The data of the present research showed that seasonal changes as well as climatic and human activities can be effective on the amount of chemical parameters of water resources.

## 1 Data

**Fig. 1:**
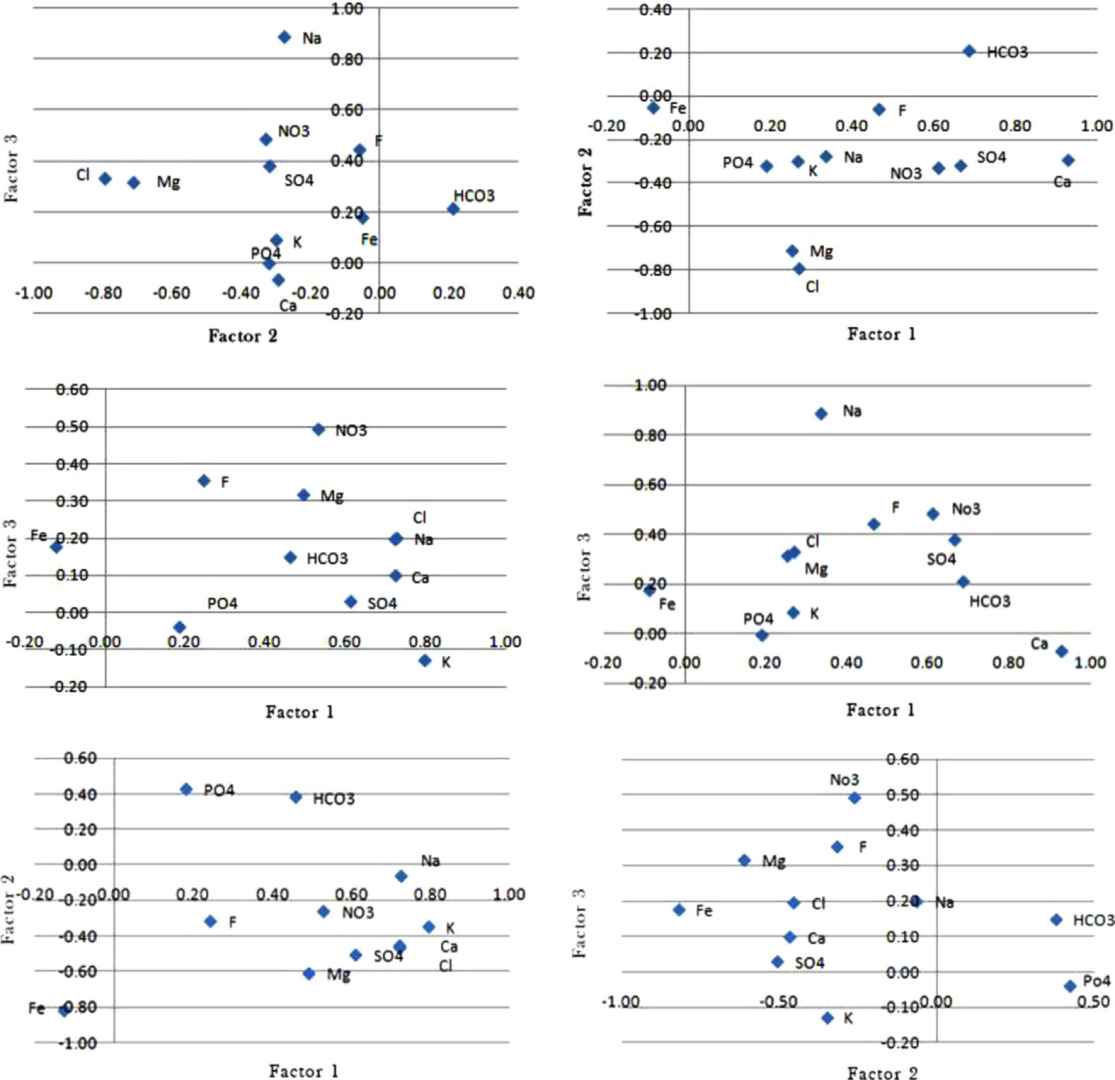
The X and Y charts of the operational data drawn in the first half of 2017.

**Fig. 2:**
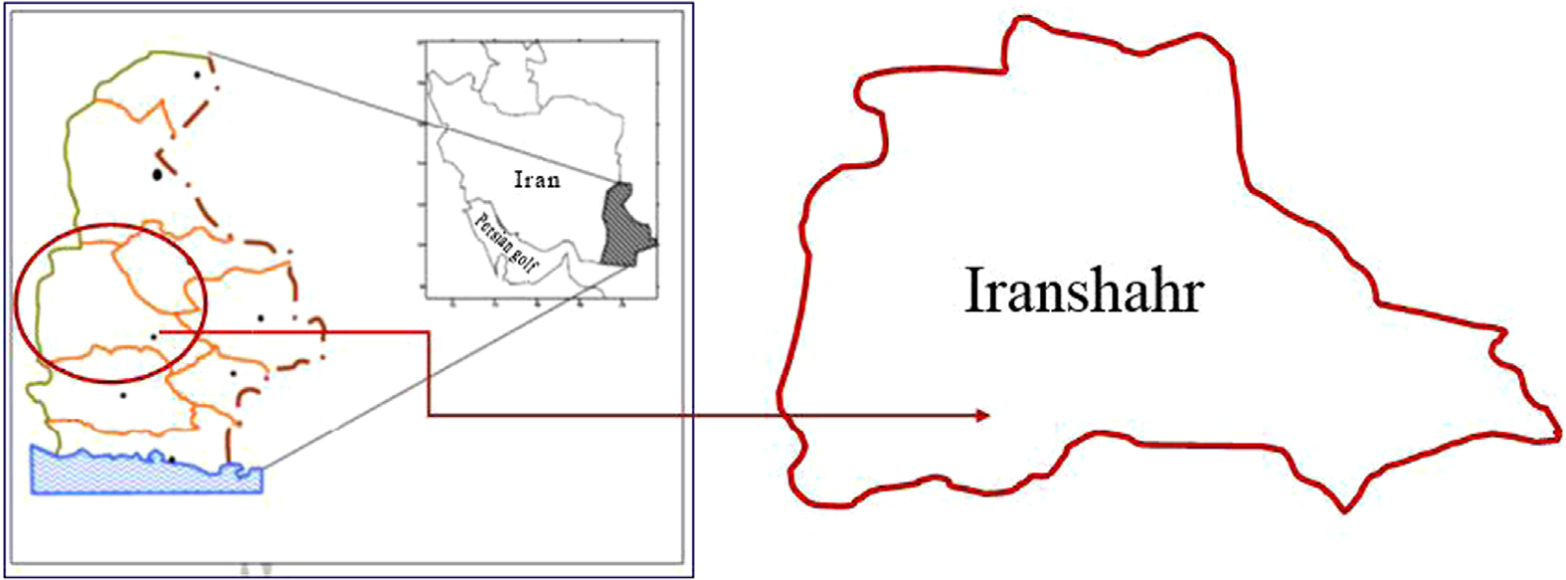
Iranshahr location in the Sistan and Baluchistan, Iran.

**Fig. 3:**
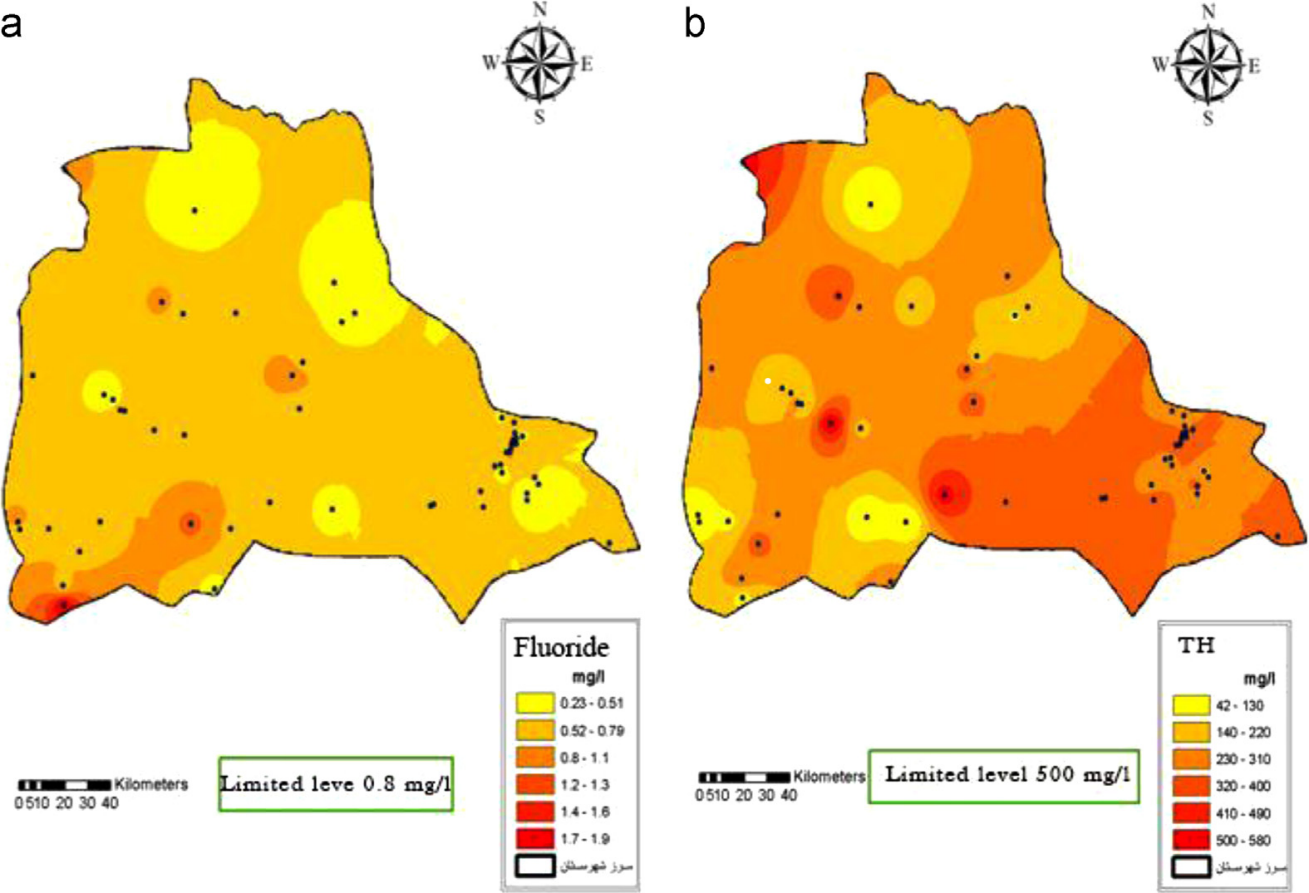
Fluoride (a) and (b) Total hardness distribution in Iranshahr groundwater׳s, 2017.

**Fig. 4:**
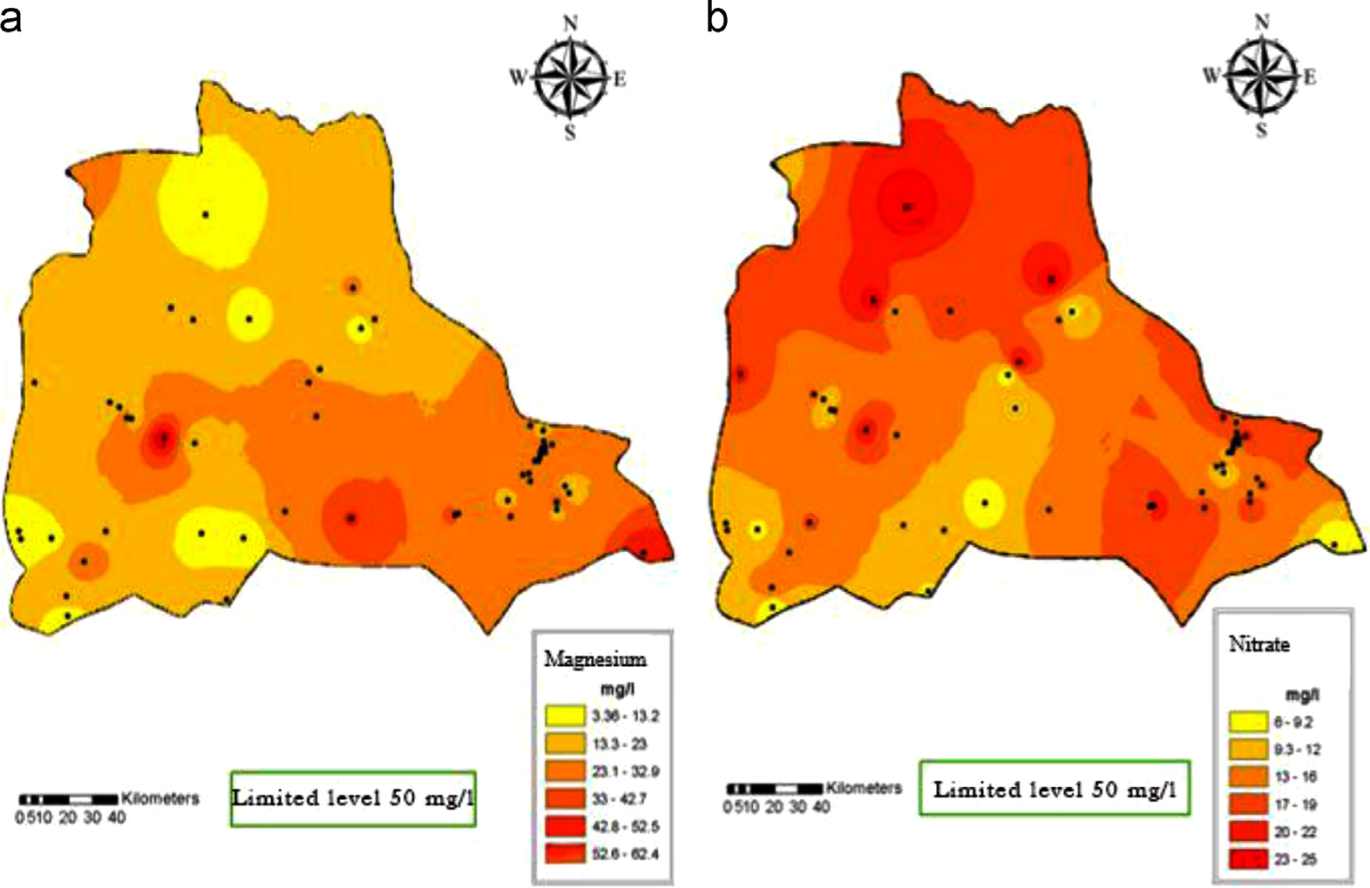
Magnesium (a) and Nitrate (b) distribution in Iranshahr groundwater׳s, 2017.

**Fig. 5:**
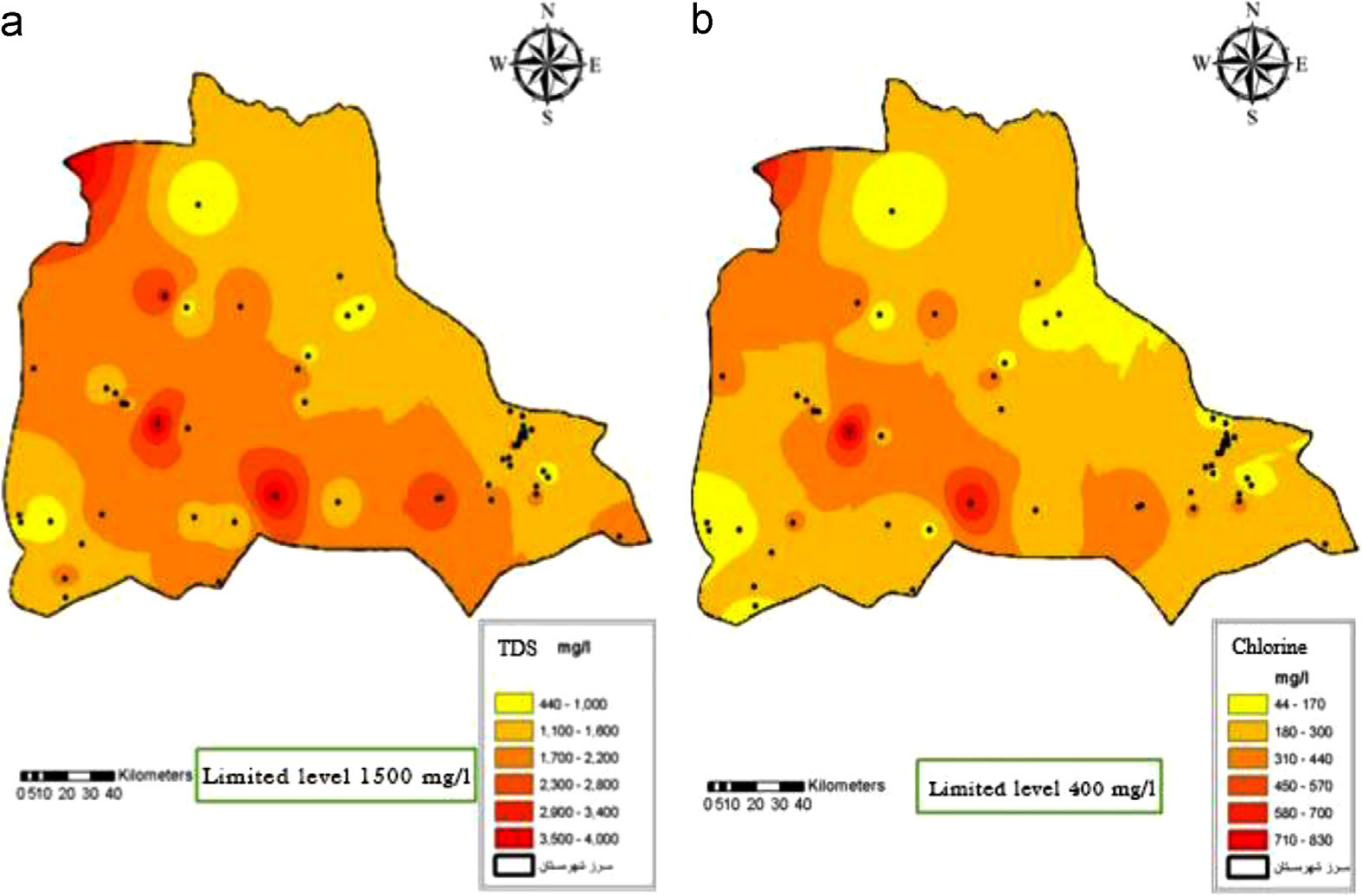
Total Dissolve Solid (a) and Chlorine (b) distribution in Iranshahr groundwater׳s, 2017.

**Fig. 6:**
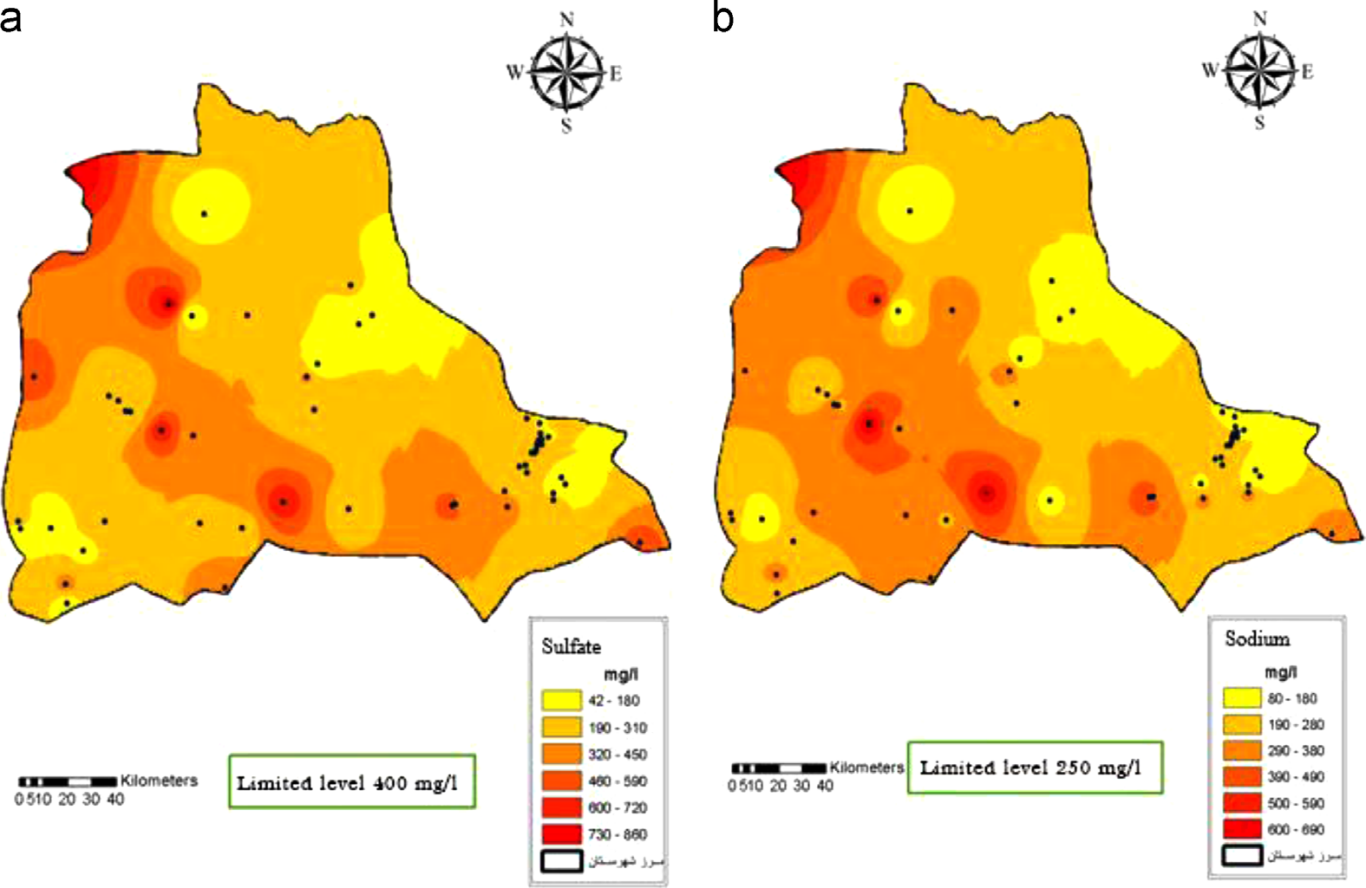
Sulfate (a) and Sodium (b) distribution in Iranshahr groundwater׳s, 2017.

**Fig. 7:**
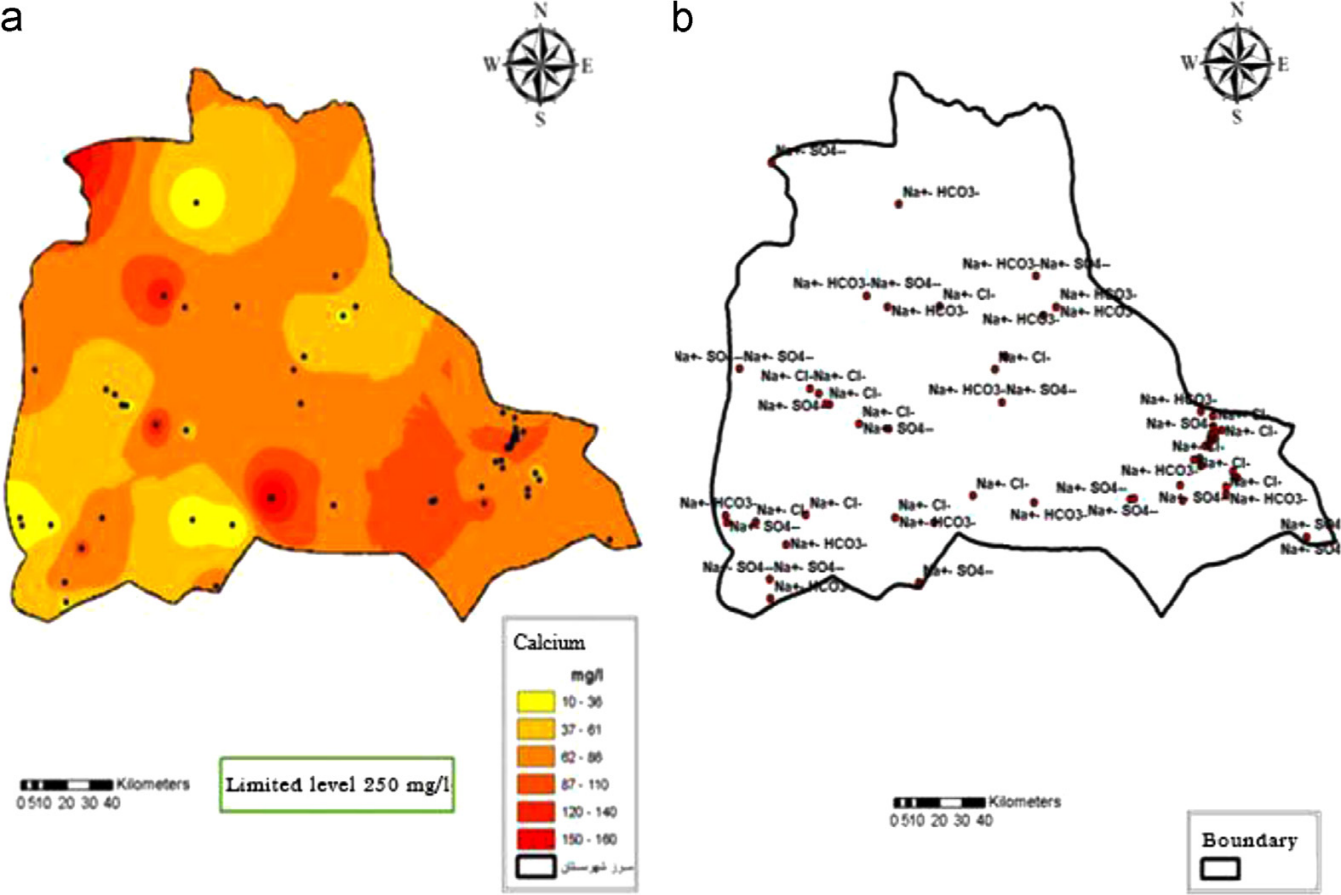
Calcium (a) and Water type (b) distribution in Iranshahr groundwater׳s, 2017.

**Fig. 8:**
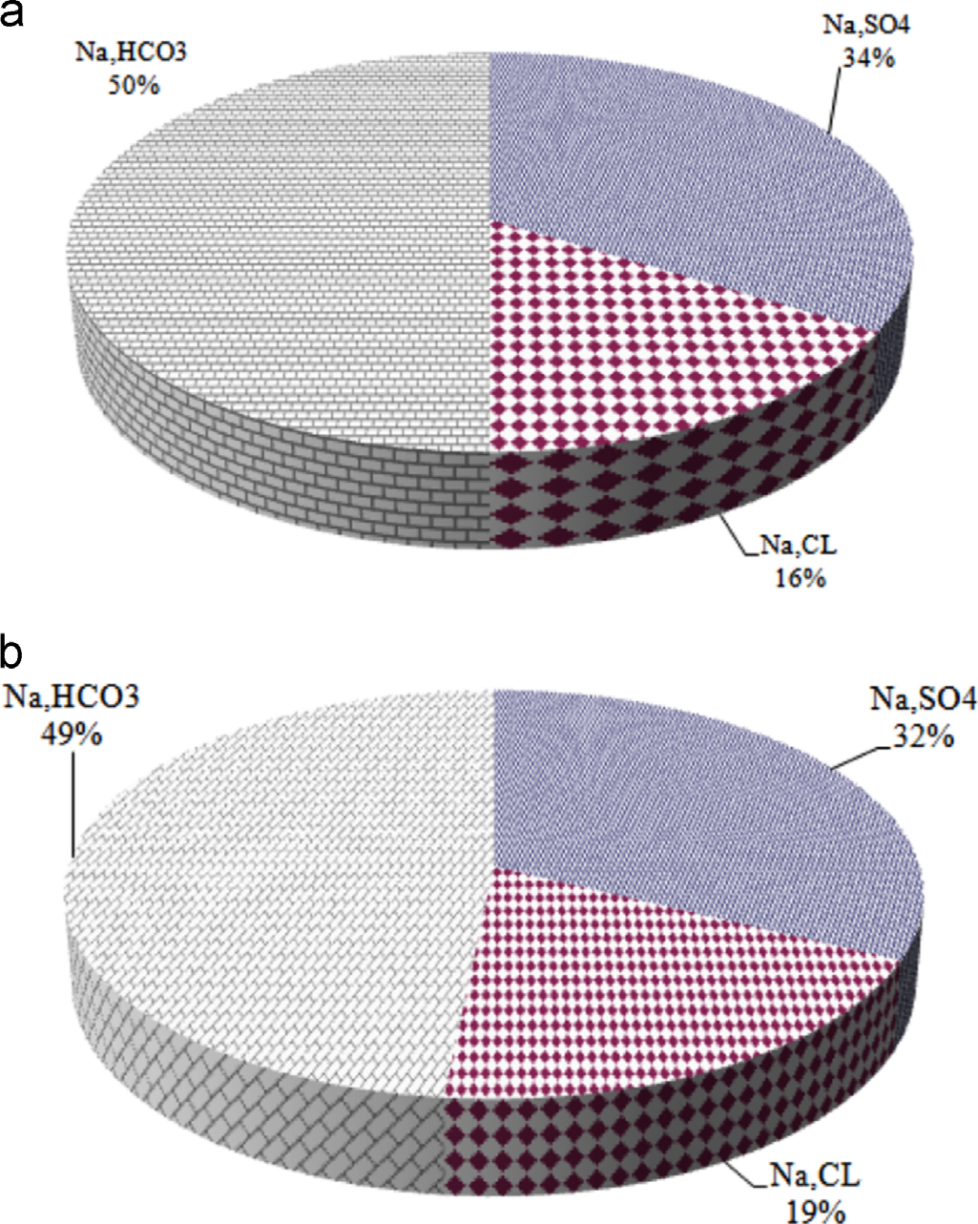
Water type in groundwater resources at the first (a) and second half of seasons, 2017.

Based on the factor model used for three factors and eleven variables, the factor 1 can be attributed to chemical processes such as chemical weathering, dissolution and ion exchange as most cations and anions have high factor load. The factor 2 can be attributed to feeding through the river and surface waters which increases the concentration of bicarbonate and phosphate in groundwater. Since nitrate concentration in groundwater is more influenced by urban sewage and agricultural fertilizer leaching, Nitrate indicates the highest factor load in the third factor and in the second half of the year. On the other hand, the concentration of sodium and chlorine has a significant increase. The factor 3 can be related to human activities, such as the influx of wastewater, absorbent wells of domestic wastewater and etc. Based on correlation coefficient in the first half of the year between factors 1 and 2 and chemical variables other than bicarbonate and phosphate (surface waters of bicarbonate which contain Phosphatic agricultural fertilizers), all chemical variables have a negative relationship with factor 2 and the only ion which has a negative relationship with factor 1 (geochemical processes of aquifer), is iron. Based on the negative relation with factors 1, it is pointed out that Iron ion has been less affected by the geochemical activity of the aquifer. However, iron ion has a positive correlation with factor 3 (human activities). Phosphate, potassium and calcium ions show a negative correlation with factor 3. Phosphate and bicarbonate ions have a positive correlation with factors 2 and 3. Due to the matter that the rate of the sodium and chlorine ions were high in the city of Iranshahr and since these ions have a high correlation with fluoride ions, it is recommended that, special attention should be paid to health issues in this regard. according to the fact that the main part of groundwater contamination in this basin is made through human activities such as the construction of absorbing wells of wastewater and the use of chemical fertilizers, etc., more desirable options are to be used in such cases so that it can be diligent to improve the quality and maintain these resources as good as possible. The results outcoming from data analysis illustrate in Table 1, Table 2, Table 3, Table 4, Table 5, Table 6, Table 7 and Fig. 1, Fig. 2, Fig. 3, Fig. 4, Fig. 5, Fig. 6, Fig. 7, Fig. 8 at following.

**Table 1:** The correlation of each ions with other ions in spring and summer, 2017.

**Correlation in the spring and summer, 2017**				**1**	**2**	**3**	**4**	**5**	**6**	**7**	**8**	**9**	**10**	**11**	**12**	**13**
**Num**	**Min**	**Max**	**Ions**	**EC**	**TDS**	**F**^**−**^	**CL**^**−**^	**SO**_**4**_^−**2**^	**HCO**_**3**_^**−**^	**NO**_**3**_^**−**^	**PO**_**4**_^**−3**^	**Ca**^**+2**^	**Mg**^**+2**^	**Na**^**+**^	**K**^**+**^	**Fe**^**+2**^
1	1	1	**EC**	1												
2	1	1	**TDS**	1	1											
3	0.34	0.61	**F**^**−**^	0.34	0.61	1										
4	0.58	0.98	**CL**^**−**^	0.98	0.98	0.58	1									
5	0.44	0.78	**SO**_**4**_^**−2**^	0.78	0.78	0.44	0.72	1								
6	0.01	0.39	**HCO**_**3**_^**−**^	0.39	0.39	0.35	0.34	0.01	1							
7	−0.09	0.17	**NO**_**3**_^**−**^	0.12	0.12	−0.09	0.13	0.17	−0.08	1						
8	−0.11	0.43	**PO**_**4**_^**−3**^	0.16	0.16	0.43	0.15	0.07	0.19	−0.11	1					
9	0.01	0.68	**Ca**^**+2**^	0.64	0.64	0.20	0.68	0.31	0.42	0.19	0.01	1				
10	−0.10	0.59	**Mg**^**+2**^	0.53	0.53	0.32	0.51	0.44	0.59	−0.10	0.16	0.41	1			
11	0.10	0.97	**Na**^**+**^	0.97	0.97	0.66	0.96	0.71	0.31	0.10	0.19	0.59	0.37	1		
12	−0.07	0.55	**K**^**+**^	0.35	0.35	0.04	0.32	0.55	-0.07	0.05	0.06	0.14	0.40	0.22	1	
13	−0.19	0.25	**Fe**^**+2**^	−0.01	−0.01	−0.02	−0.01	−0.03	0.24	−0.11	−0.19	0.11	0.25	−0.10	0.10	1
The average of rows and columns for each ion				4.12	4.26	2.93	4.17	3.47	2.54	1.19	1.64	3.17	3.20	3.98	2.26	1.11

**Table 2:** The correlation of each ions with other ions in spring and summer, 2017.

**Correlation in the autumn and winter, 2010**				**1**	**2**	**3**	**4**	**5**	**6**	**7**	**8**	**9**	**10**	**11**	**12**	**13**
**Num**	**Max**	**Min**	**Ions**	**EC**	**TDS**	**F**^**−**^	**CL**^**−**^	**SO**_**4**_^**−2**^	**HCO**_**3**_^**−**^	**NO**_**3**_^**−**^	**PO**_**4**_^**−3**^	**Ca**^**+2**^	**Mg**^**+2**^	**Na**^**+**^	**K**^**+**^	**Fe**^**+2**^
1	1	1	**EC**	1												
2	1	1	**TDS**	1	1											
3	0.3	140.14/0	**F**^**−**^	0.14	0.30	1										
4	0.97	0.25	**CL**^**−**^	0.97	0.97	0.25	1									
5	0.96	0.26	**SO**_**4**_^**−2**^	0.96	0.96	0.26	0.89	1								
6	0.34	0.07	**HCO**_**3**_^**−**^	0.23	0.23	0.34	0.07	0.19	1							
7	0.37	−0.05	**NO**_**3**_^**−**^	0.32	0.32	0.01	0.30	0.37	−0.05	1						
8	0.26	−0.13	**PO**_**4**_^**−3**^	0.20	0.20	0.10	0.20	0.24	−0.31	0.26	1					
9	0.54	−0.13	**Ca**^**+2**^	0.54	0.54	−0.13	0.50	0.51	0.31	0.29	0	1				
10	0.83	−0.08	**Mg**^**+2**^	0.64	0.64	0.20	0.59	0.59	0.43	0.31	−0.08	0.83	1			
11	0.96	0.19	**Na**^**+**^	0.96	0.96	0.40	0.93	0.93	0.19	0.29	0.22	0.30	0.44	1		
12	0.59	−0.2	**K**^**+**^	0.36	0.36	−0.07	0.34	0.38	−0.20	0.34	0.59	0.46	0.19	0.26	1	
13	0.19	−0.01	**Fe**^**+2**^	0.05	0.05	0.02	0.05	0.04	−0.01	0.07	0.05	0.07	0.19	0.03	0.06	1
The average of rows and columns for each ion				4.18	4.26	1.82	4.03	4.16	1.71	2.41	1.83	3.11	3.96	3.96	2.53	1.33

**Table 3:** Maximum, minimum, mean, standard deviation and out of range for each element in Iranshahr, 2017.

**Elemental elements**	Fe^+2^	K^+^	Na^+^	Mg^+2^	Ca^+2^	PO_4_^−3^	NO_3_^−^	HCO_3_^−^	SO_4_^−2^	CL^−^	F^−^	TDS	EC
**The values of chemical parameters measured in groundwater resources of Iranshahr during the first half of 2017**													
**Maximum**	0.024	12	10.65	39.36	480.80	0.36	28	431.88	880	954	1.72	3078	4810
**medium**	0.011	4.54	269.83	17.24	72.87	0.11	14.62	240.07	247.74	245.16	0.63	1038.93	1623.54
**minimum**	0.000	2	65	4.8	16	0.04	6.16	102.48	40	39.70	0.25	276	432
**Standard deviation**	0.005	2.20	190.38	8.43	62.09	0.05	4.68	83.36	154.18	177.94	0.26	567.05	886
**Out of range (%)**	0	–	59.68	0	1.61	–	0	–	16.13	9.68	22.58	11.29	–

**The values of chemical parameters measured in groundwater resources of Iranshahr during the second half of 2017**													
**Maximum**	0.09	30	997	67.68	155.20	0.61	30	400.16	1000	883	1.18	3130	4890
**medium**	0.02	5.46	284.90	21.07	65.17	0.155	14.62	242.28	277.41	270.32	0.46	1060.01	1657.40
**minimum**	0	1	5	1.92	11.20	0	7	85.4	50	35	0.10	241	376
**Standard deviation**	0.017	4.92	182.14	14.56	36.38	0.108	5.57	76.93	204.93	182.42	0.22	578.99	903.43
**Out of range (%)**	0	–	67.74	3.32	0	–	0	–	20.97	12.90	9.06	12.90	–

**Table 4:** The result of subtracting the sum of each row and column for each parameter in order to determine the rate of progression or reversal of the correlation matrix and the relationship between the parameters by passing the warm seasons to the cold seasons, 2017.

**Total First Six Months − Total Six Months = The relationship between ions**				**1**	**2**	**3**	**4**	**5**	**6**	**7**	**8**	**9**	**10**	**11**	**12**	**13**
**Num**	**Min**	**Max**	**Ions**	**EC**	**TDS**	**F**^**−**^	**CL**^**−**^	**SO**_**4**_^**−2**^	**HCO**_**3**_^**−**^	**NO**_**3**_^**−**^	**PO**_**4**_^**−3**^	**Ca**^**+2**^	**Mg**^**+2**^	**Na**^**+**^	**K**^**+**^	**Fe**^**+2**^
1	0	0	**EC**	0												
2	0	0	**TDS**	0	0											
3	−0.33	0	**F**^**−**^	−0.20	−0.30	0										
4	−0.33	0	**CL**^**−**^	−0.01	−0.01	−0.33	0									
5	−0.18	0.18	**SO**_**4**_^**−2**^	0.18	0.18	−0.18	0.17	0								
6	−0.27	0.18	**HCO**_**3**_^**−**^	−0.16	−0.16	−0.01	−0.27	0	0							
7	0	0.2	**NO**_**3**_^−^	0.20	0.20	0.10	0.17	0.2	0.03	0						
8	−0.5	0.37	**PO**_**4**_^**−3**^	0.04	0.04	−0.33	0.05	0.17	−0.50	0.37	0					
9	−0.33	0.20	**Ca**^**+2**^	−0.10	−0.10	−0.33	−.0.18	0.20	−0.11	0.10	−0.01	0				
10	−0.30	0.42	**Mg**^**+2**^	0.11	0.11	−0.30	0.08	0.15	−0.16	0.41	−0.24	0.42	0			
11	−0.29	0.22	**Na**^**+**^	−0.01	−0.01	−0.26	−0.33	0.22	−0.29	0.19	0.03	−0.29	0.07	0		
12	−0.21	0.53	**K**^**+**^	0.10	0.01	−0.11	0.01	−0.17	−0.13	0.29	0.53	0.23	−0.21	−0.4	0	
13	−0.25	0.24	**Fe**^**+2**^	0.06	0.06	0.04	0.06	0.07	−0.25	0.18	0.24	−0.04	−0.06	0.13	−0.04	0

**Table 5:** The values of each factor along with the presentation of the relationship between them.

**Min**	**Max**	**Variables**	**First factor**	**Second factor**	**Third factor**	**Subscription rate**
**The operating pattern obtained for groundwater resources of Iranshahr city in the first six months, 2017**						
−0.293	0.930	**F**^**−**^	0.930	−0.293	−0.070	0.996
−0.718	0.313	**CL**^**−**^	0.253	−0.710	0.330	0.633
−0.276	0.886	**SO**_**4**_^**−2**^	0.336	−.276	0.886	0.953
−0.299	0.276	**HCO**_**3**_^**−**^	0.276	−0.299	0.086	0.210
0.210	0.687	**NO**_**3**_^**−**^	0.678	0.210	0.210	0.489
−0.792	0.329	**PO**_**4**_^**−3**^	0.270	−0.792	0.329	1
−0.318	0.666	**Ca**^**+2**^	0.666	−0.318	0.378	0.618
−0.329	0.612	**Mg**^**+2**^	0.612	−0.330	0.483	0.597
−0.320	0.190	**Na**^**+**^	0.190	−0.320	−0.005	0.236
−0.059	0.466	**K**^**+**^	0.466	−0.059	0.442	0.535
0.088	0.175	**Fe**^**+2**^	−0.088	−0.051	0.175	0.080

**The operating pattern obtained for groundwater resources of Iranshahr city in the second six months, 2017**						
−0.446	0.724	**F**^**−**^	0.724	−0.466	0.09	0.733
−0.610	0.494	**CL**^**−**^	0.494	−0.610	0.316	0.836
−0.063	0.727	**SO**_**4**_^**−2**^	0.728	−0.063	0.199	0.636
−0.347	0.797	**HCO**_**3**_^**−**^	0.797	−0.347	−0.129	0.682
0.148	0.461	**NO**_**3**_^**−**^	0.461	0.379	0.148	0.431
−0.454	0.723	**PO**_**4**_^**−3**^	0.723	−0.454	0.196	0.676
−0.505	0.612	**Ca**^**+2**^	0.612	−.505	0.029	0.607
−0.261	0.531	**Mg**^**+2**^	0.513	−0.261	0.492	0.734
−0.040	0.432	**Na**^**+**^	0.184	0.424	−0.042	0.716
−0.316	0.354	**K**^**+**^	0.254	−0.316	0.354	0.776
−0.817	0.176	**Fe**^**+2**^	−0.124	−0.817	0.176	0.763

**Table 6:** Testing the *t*-value of the values of r obtained to determine whether there is a correlation or acceptable relation between the variables.

**Grayscale points show that there is a correlation and acceptable relation between the parameters with the assumption of alpha α 0.05 and t (2.6877) in the first six months of 2017**																
$t=r\sqrt{\frac{n-2}{1-r^{2}}}$				**1**	**2**	**3**	**4**	**5**	**6**	**7**	**8**	**9**	**10**	**11**	**12**	**13**
**Num**	**Min**	**Max**	**Ions**	**EC**	**TDS**	**F**^**−**^	**CL**^**−**^	**SO**_**4**_^**−2**^	**HCO**_**3**_^**−**^	**NO**_**3**_^**−**^	**PO**_**4**_^**−3**^	**Ca**^**+2**^	**Mg**^**+2**^	**Na**^**+**^	**K**^**+**^	**Fe**^**+2**^
1	*	*	**EC**	*												
2	*	*	**TDS**	*	*											
3	2.80	5.96	**F**^**−**^	2.80	5.96	*										
4	5.51	38.14	**CL**^**−**^	38.14	38.14	5.51	*									
5	3.79	9.65	**SO**_**4**_^**−2**^	9.65	9.65	3.79	8.03	*								
6	0.077	3.28	**HCO**_**3**_^**−**^	3.28	3.28	2.89	2.80	0.077	*							
7	−0.70	1.33	**NO**_**3**_^**−**^	0.93	0.93	−0.70	1.01	1.33	-0.62	*						
8	−0.85	3.68	**PO**_**4**_^**−3**^	1.25	1.25	3.69	1.17	0.54	1.49	−0.85	*					
9	0.077	7.18	**Ca**^**+2**^	6.45	6.45	1.58	7.18	2.52	3.58	1.49	0.077	*				
10	−0.778	5.66	**Mg**^**+2**^	4.84	4.84	2.61	4.59	3.79	5.66	−0.77	1.25	3.48	*			
11	0.778	30.90	**Na**^**+**^	30.90	30.90	6.80	26.55	7.81	2.52	0.77	1.49	5.66	3.08	*		
12	−0.54	5.10	**K**^**+**^	2.89	2.89	0.31	2.70	5.10	−0.54	0.38	0.46	1.09	3.38	1.74	*	
13	−1.49	2.00	**Fe**^**+2**^	−0.977	−0.077	−0.15	−0.077	−0.23	1.91	−0.85	−1.49	0.85	2.00	−0.77	0.77	*

^*^ The correlation between these parameters it were 100 percent, so t-factor for these parameters is not calculate.

**Table 7:** Testing the values of *t*-factors relative to “*r*” values obtained to determine whether there is a correlation or acceptable relation between variables.

**Grayscale points show that there is a correlation and acceptable relation between the parameters with the assumption of alpha α 0.05 and t (2.6877) in the second six months of 2017**																
$t=r\sqrt{\frac{n-2}{1-r^{2}}}$				**1**	**2**	**3**	**4**	**5**	**6**	**7**	**8**	**9**	**10**	**11**	**12**	**13**
**Num**	**Min**	**Max**	**Ions**	**EC**	**TDS**	**F**^**−**^	**CL**^**−**^	**SO**_**4**_^**−2**^	**HCO**_**3**_^**−**^	**NO**_**3**_^**−**^	**PO**_**4**_^**−3**^	**Ca**^**+2**^	**Mg**^**+2**^	**Na**^**+**^	**K**^**+**^	**Fe**^**+2**^
1	*	*	**EC**	*												
2	*	*	**TDS**	*	*											
3	1.09	2.43	**F**^**−**^	1.09	2.43	*										
4	2.00	30.90	**CL**^**−**^	30.90	30.90	2.00	*									
5	2.08	26.55	**SO**_**4**_^**−2**^	26.55	26.55	2.08	15.12	*								
6	0.54	2.80	**HCO**_**3**_^**−**^	1.83	1.83	2.80	0.54	1.49	*							
7	−0.38	3.08	**NO**_**3**_^**−**^	2.61	2.61	0.077	2.43	3.08	−0.38	*						
8	−2.52	2.08	**PO**_**4**_^**−3**^	1.58	1.58	0.77	1.58	1.91	−2.52	2.08	*					
9	−1.01	4.97	**Ca**^**+2**^	4.97	4.97	−1.01	4.47	4.50	2.52	2.34	0.00	*				
10	−0.62	11.52	**Mg**^**+2**^	6.45	6.45	0.15	5.66	5.66	3.68	2.52	−0.62	11.52	*			
11	1.49	26.55	**Na**^**+**^	26.55	26.55	3.38	19.59	19.59	1.49	2.34	1.74	2.43	3.79	*		
12	−1.58	5.66	**K**^**+**^	2.98	2.98	−0.54	2.80	3.18	−1.58	2.80	5.66	4.01	1.49	2.08	*	
13	−0.77	1.49	**Fe**^**+2**^	0.38	0.38	0.15	0.38	0.31	−0.077	0.54	0.38	0.54	1.49	0.23	0.46	*

^*^ The correlation between these parameters it were 100 percent, so t-factor for these parameters is not calculate.

## 2 Experimental design, materials and methods

### 2.1 Method of implementation

The current descriptive and analytical study was conducted from April 17, 2017 to March 24, 2017.The studied population included underground water resources in the villages of Iranshahr city and the sampling site was chosen according to our previous studies from the line of harvest to the nearest part of the water resource in the desired area [1]. Number of samples taken from each source was a total of 248 samples (with two-time repetitions in one-week intervals) from underground water sources in different locations of Iranshahr city by a random cluster sampling method considering the size of the community.

The experiments of measuring 13 chemical parameters were divided into two general categories including device-based experiments and Titrimetry tests. They were also carried out according to the reference book for water and wastewater testing and other valid references [2], [3], [4], [5], [6], [7], [8], [9], [10], [11], [12], [13], [14], [15], [16].

Determination of temporal and permanent values of hardness, calcium and magnesium, iron, nitrate, alkalinity, sulfate and chloride by titrimetry and alkalinity by titration with Chloride acid or 0.02*n* sulfuric acid were carried out according to the methods stated in the method standard reference [17], [18], [19], [20], [21], [22], [23], [24].

### 2.2 Instrument

Device-based experiments including the EC and TDS measurements were performed by EC-meter device (CD20 model) with the Aqualytic symbol and precision of 0.01. The measurement device was made in Germany.

Other anions and cations were measured using the T80 UV Visible spectrophotometer. Finally, the dispersion of chemical elements was also plotted in the city by the GIS system [21]. The dominant water type in the groundwater resources of this city based on determining the component of the largest amount of cation among the cations and the largest amount of anion among other anions was determined and reported by conditional function in Microsoft Excel and during two warm and cold seasons with 124 samples which were repeated twice in each resource.

To determine the hydro-geochemical characteristics of Iranshahr water basin and identification of factors and their impact on the combination of groundwater, factor analysis method was used which has three stages consisting of developing a correlation matrix of all variables, extracting factors and interpreting the results on the basis of correlation matrix and Pearson correlation coefficient [25].

### 2.3 Analytical methods

To examine the correlation between variables (The Observational variables used in hydro-geochemical studies are the results of chemical analysis of water), it should be considered that distribution of an attribute in terms of the different values of the second attribute, in general, cannot determine the method of second attribute distribution in terms of this attribute and the type of relationship between the two attributes is possible by having their dual distributions or selection of random sample of this distribution.

The correlation analysis formula was used as the Eq. (1) to determine the adherence of any attribute from another attribute. In the above-mentioned equation, *n* is referred to the number of data, Xi and Yi indicate each of the values of the variables [26].

(1) $$r=\frac{\left(\sum XiYi-\sum Xi\sum Yi\right)}{\sqrt{\left[n\sum Xi^{2}-{\left(\sum Xi\right)}^{2}\right]\left[n\sum Yi^{2}-{\left(\sum Yi\right)}^{2}\right]}}$$

Finally, the factors were determined according to the standard method of factor analysis (actually, these factors reflect the mechanisms affecting the composition of groundwater).then the origin of each of these factors was interpreted and determined based on factor loads (the correlation of each variable with each factor is called factor load), hydrogeological, geological and hydro-chemical conditions [1], [27].
