# Supplementary material for: Data for factor analysis of hydro-geochemical characteristics of groundwater resources in Iranshahr
Source: Data Brief. 2018 May 18;19:548–63. doi: 10.1016/j.dib.2018.05.039 (PMC5997880; doi:10.1016/j.dib.2018.05.039)
Supplement: Supplementary file 1 — Transparency document. [file mmc1.docx]

Dear editor(s)

Data in Brief

Greetings!

At this time, this article with entitled”***Data for Factor Analysis of Hydro-geochemical Characteristics of Groundwater Resources in Iranshahr “***has not been published or submitted in any other journals. We hope that the results of this research will be of interest to you. None of the authors of this paper have any conflicts of interest to declare.

Sincerely yours

Corresponding author

Hooshmand Sharafi
